# Psychological risk factors that characterize the trajectories of quality of life after a physical trauma: a longitudinal study using latent class analysis

**DOI:** 10.1007/s11136-020-02740-x

**Published:** 2021-01-14

**Authors:** Eva Visser, Brenda Leontine Den Oudsten, Taco Gosens, Paul Lodder, Jolanda De Vries

**Affiliations:** 1 Department Trauma TopCare, ETZ Hospital (Elisabeth-TweeSteden Ziekenhuis), Tilburg, The Netherlands; 2 Department of Medical and Clinical Psychology, Tilburg University, Warandelaan 2, 5037 AB Tilburg, The Netherlands; 3 Department of Orthopaedics, ETZ Hospital (Elisabeth-TweeSteden Ziekenhuis), Tilburg, The Netherlands; 4 Department of Methodology and Statistics, Tilburg University, Tilburg, The Netherlands

**Keywords:** Quality of life, Trauma, Injury, Repeated measures latent class analysis, Observational cohort research

## Abstract

**Background:**

The course and corresponding characteristics of quality of life (QOL) domains in trauma population are unclear. Our aim was to identify longitudinal QOL trajectories and determine and predict the sociodemographic, clinical, and psychological characteristics of trajectory membership in physical trauma patients using a biopsychosocial approach.

**Methods:**

Patients completed a questionnaire set after inclusion, and at 3, 6, 9, and 12 months follow-up. Trajectories were identified using repeated-measures latent class analysis. The trajectory characteristics were ranked using Cohen’s d effect size or phi coefficient.

**Results:**

Altogether, 267 patients were included. The mean age was 54.1 (SD = 16.1), 62% were male, and the median injury severity score was 5.0 [2.0—9.0]. Four latent trajectories were found for psychological health and environment, five for physical health and social relationships, and seven trajectories were found for overall QOL and general health. The trajectories seemed to remain stable over time. For each QOL domain, the identified trajectories differed significantly in terms of anxiety, depressive symptoms, acute stress disorder, post-traumatic stress disorder, Neuroticism, trait anxiety, Extraversion, and Conscientiousness.

**Discussion:**

Psychological factors characterized the trajectories during 12 months after trauma. Health care providers can use these findings to identify patients at risk for impaired QOL and offer patient-centered care to improve QOL.

**Supplementary information:**

The online version of this article (10.1007/s11136-020-02740-x) contains supplementary material, which is available to authorized users.

## Introduction

Physical trauma became a major public health problem over the last decade, because an increasing number of patients were treated in the emergency department (ED) after injury [1]. Survivorship increased due to improvement in specialized trauma care [2]. Nevertheless, survivors have reported long-term physical disabilities (e.g., pain and fatigue), psychological problems (e.g., anxiety and depressive symptoms), disorders (e.g., acute and post-traumatic stress disorder (PTSD)) [3–8], and impaired quality of life (QOL; i.e., a subjective and multidimensional concept of person's physical health, psychological state, personal beliefs, social relationships and their relationship to salient features of their environment) [9–13].

These disabilities and disorders were, together with sociodemographic (e.g., older age, female sex, low education) and clinical (e.g., higher injury severity score, hospital stay and ICU admission) characteristics, related to impaired health-related QOL (HRQOL) or health status (HS) [9, 12, 14–18]. HRQOL is a limited definition of QOL, as it solely focuses on patients’ subjective perceptions on health (i.e., physical and mental health), whereas HS refers to the extent of physical, psychological, and social functioning, but without taken patients’ satisfaction with functioning into account [19]. Moreover, recent studies, describing latent trajectories, focused on general health [20] and health status (HS) [21–23] and not on QOL. These studies were also based on a subset of the trauma population (e.g., whiplash or traumatic brain injury), instead of a trauma population with multiple injuries.

To our knowledge, no study has been conducted to identify trajectories and predictors for impaired QOL after injury. Repeated-measures latent class analysis (RMLCA) can be used to identify a set of distinct longitudinal response patterns (i.e., QOL trajectories). Regression analyses can subsequently be used to examine the sociodemographic and clinical characteristics of patients classified in each trajectory [24]. Therefore, our aims were to first identify latent trajectories representing distinct changes in QOL over a 12-month follow-up and then to determine the sociodemographic, clinical, and psychological characteristics of each identified trajectory using a biopsychosocial approach [25].

## Methods

### Patients

Trauma patients treated in the shock room between November 2016 and November 2017 of the ETZ Hospital (Elisabeth-TweeSteden Ziekenhuis), Tilburg, The Netherlands, were asked to participate in this study. This hospital is a Level-1 Trauma Center in the province of Noord-Brabant. Only patients aged 18 or older were included. Patients were excluded in case of severe traumatic brain injury (i.e., Glasgow Coma Score (GCS) ≤ 8), dementia, or insufficient knowledge of the Dutch language (verbal and writing).

### Study design and procedure

Patients were asked to participate by either the emergency doctor or the researcher (EV). Patients signed two informed consents: first, in the emergency department after receiving treatment in the shock room and being informed by the doctor; then 1 to 5 days later, patients again confirmed participation to make sure that they have had sufficient time to consider participation in the study. Unconscious patients were informed by the researcher and asked to participate as soon as they were lucid. All obtained information was destroyed for patients who declined participation by not signing the second informed consent.

This study is part of a mixed-method study. The study protocol is published elsewhere [26]. This study (protocol number: NL55386.028.15) has been reviewed and approved by the Medical Ethical Committee Brabant (METC Brabant) on December 4, 2015. The study has been recorded in the Netherlands Trial Registry (NTR6258). To strengthen validity and comprehensiveness, this study was conducted and reported according to the Strengthening the Reporting of Observational Studies in Epidemiology (STROBE) checklist for cohort studies [27]. Participation was voluntarily and no financial reward was given.

### Data collection

Sociodemographic information (i.e., sex, age, living situation, education level, and employment) was obtained from patients at baseline. Clinical information, including type of trauma mechanism (e.g., motor vehicle accident), number of injuries, type of injury (e.g., spinal cord injury), injury severity score (ISS), GCS, surgery (yes/no), hospital admission (yes/no), admission to ICU, length of stay, psychiatric history (yes/no), and consult or treatment from health psychologist (yes/no), was abstracted from the patients’ medical records.

Data for this study were collected using self-report questionnaires and a structured interview. Patients completed a baseline questionnaire on sociodemographics, QOL, ASD and PTSD, anxiety, depressive symptoms, and personality traits after they confirmed participation. Clinical information was retrieved from patients’ medical records. QOL was further assessed during follow-up at 3, 6, 9, and 12 months after injury [26].

QOL was measured with the World Health Organization Quality of Life assessment instrument-Bref (WHOQOL-Bref) [25, 28]. This 26-item questionnaire is the short version of the WHOQOL-100 and assesses four domains (Physical health, Psychological health, Social relationships, and Environment) as well as one general facet Overall QOL and general Health. Each item is rated on a five-point rating scale. Norm scores [29] were used to indicate and label each trajectory (e.g., Physical health; Poor: 9.1, Fair: 12.3, Good: 14.8, Very good: 16.5, Excellent: 18.3). Higher scores indicate better QOL. The WHOQOL-Bref has good psychometric properties [25, 30, 31] and it is a reliable and valid instrument in trauma patients [32].

The MINI-Plus is a short-structured interview, based on the Diagnostic and Statistical Manual of Mental Disorders (DSM-5), and it is used to assess ASD at baseline [33]. The items are dichotomous (symptoms: absent or present). The total scores theoretically range from 0 to 14 and indicate symptom severity. Nevertheless, patients can only be diagnosed with ASD if at least nine symptoms are present from each of the five categories (i.e., intrusion, negative emotions, dissociation, avoidance, and arousal). Therefore, in line with the manual instructions, dichotomous scores (disorder: no versus yes) for ASD were used in the analyses.

The IES-R is a self-report questionnaire to assess symptom severity of PTSD. It consists of 22 items which measure intrusive re-experiences (8 items, e.g., Any reminder brought back feelings about it’), hyperarousal (6 items, e.g., ‘I felt irritable and angry’), and avoidance (8 items, e.g., ‘I avoided letting myself get upset’) of injury-related stimuli [34]. The participant stated whether the content of each statement was present during the past 7 days on a 4-point Likert scale ranging from 0 (*not at all*) to 4 (*often*). The total scores theoretically ranged from 0 to 88 and continuous scores were used in the analyses. The IES-R has good psychometric properties [35] and the Dutch translation [36] of the IES-R is reliable and valid in various populations of people experiencing traumatic stress [37].

The Hospital Anxiety and Depression Scale (HADS) was used to measure anxiety and depressive symptoms [38]. It is a generic questionnaire measuring levels of anxiety (7 items) and depression (7 items) with a 4-point rating scale ranging from 0 (*not at all*) to 3 (*very much*). The total scores for both subscale theoretically range from 0 to 21. The questionnaire is reliable and valid in patients with traumatic brain injury [39].

The 60-item NEO Five Factor Inventory (NEO-FFI) was used to measure Big Five personality domains: Neuroticism, Extraversion, Openness to experience, Agreeableness, and Conscientiousness [40, 41]. Each of the 60 items is rated on a five-point rating scale ranging from 1 (*strongly disagree*) to 5 (*strongly agree*), resulting in domain scores theoretically ranging between 12 and 60. The psychometrics have been extensively assessed and the internal consistency, test–retest reliability, and validity are acceptable to good in physical trauma patients [42].

The State-Trait Anxiety Inventory (STAI) (short form) consists of 20 items for measuring state anxiety (10 items) and trait anxiety (10 items) [43]. In this study, only the STAI-Trait scale was used. The STAI-Trait scale has a four-point rating scale ranging from 1 (*almost never*) to 4 (*almost always*), resulting in a total score theoretically ranging from 10 to 40. The Dutch version of the STAI is a reliable and valid instrument in the general population [43].

### Statistical analysis

Missing item scores of the WHOQOL-Bref, IES-R, and the HADS were imputed with individual subscale means when at least half of the subscale items were answered [34, 44, 45].

Baseline characteristics (i.e., sociodemographic, clinical, and psychological variables) of participants versus non-participants were compared using independent t-tests for continuous normally distributed data, Mann–Whitney U tests for continuous non-normally distributed data, Chi-square tests for categorical data, and Fisher’s exact tests for categorical data (e.g., ASD) where one or more of the crosstab cells showed expected cell counts less than 5.

The software Latent Gold (version 5.1) [46] was used to conduct a RMLCA, to identify the number of non-observed (latent) trajectories in the courses of each the QOL domain scores (dependent variables). Time was modeled as a categorical predictor with five measurements, allowing for the estimation of non-linear QOL trajectories over time. Missing values on the dependent variables were handled through full information maximum likelihood estimation. The Bayesian Information Criterion (BIC) was used to determine the number of trajectories that best fitted the data, based on the rule that lower BIC values indicate better model fit [24, 47]. Class membership was determined using Latent Gold’s model class assignment procedure by assigning patients to a trajectory with the highest membership probability. The identified trajectory classes were compared on the sociodemographic, clinical and psychological characteristics using Chi-square tests and ANOVA's. As a result, each class represents a different trajectory of QOL, and each trajectory has its own characteristics. A Bonferroni–Holm correction was used to adjust the significance level for the large number of performed statistical tests [48].

For all significant (based on Bonferroni–Holm correction) continuous characteristics, Cohen’s d effect sizes were calculated to determine what characteristics are most strongly related to class membership [49]. Phi coefficients were used to examine the correlation between class membership and categorical characteristics (e.g., ASD). For each domain, three characteristics with the largest effect sizes were reported. While comparing trajectories, Good or Excellent trajectory (i.e., class with highest mean QOL scores over 12 months after injury) served as the reference class and was compared with Poor or Worse (i.e., class with lowest mean QOL scores over 12 months after trauma) QOL trajectory.

## Results

**Fig. 1:**
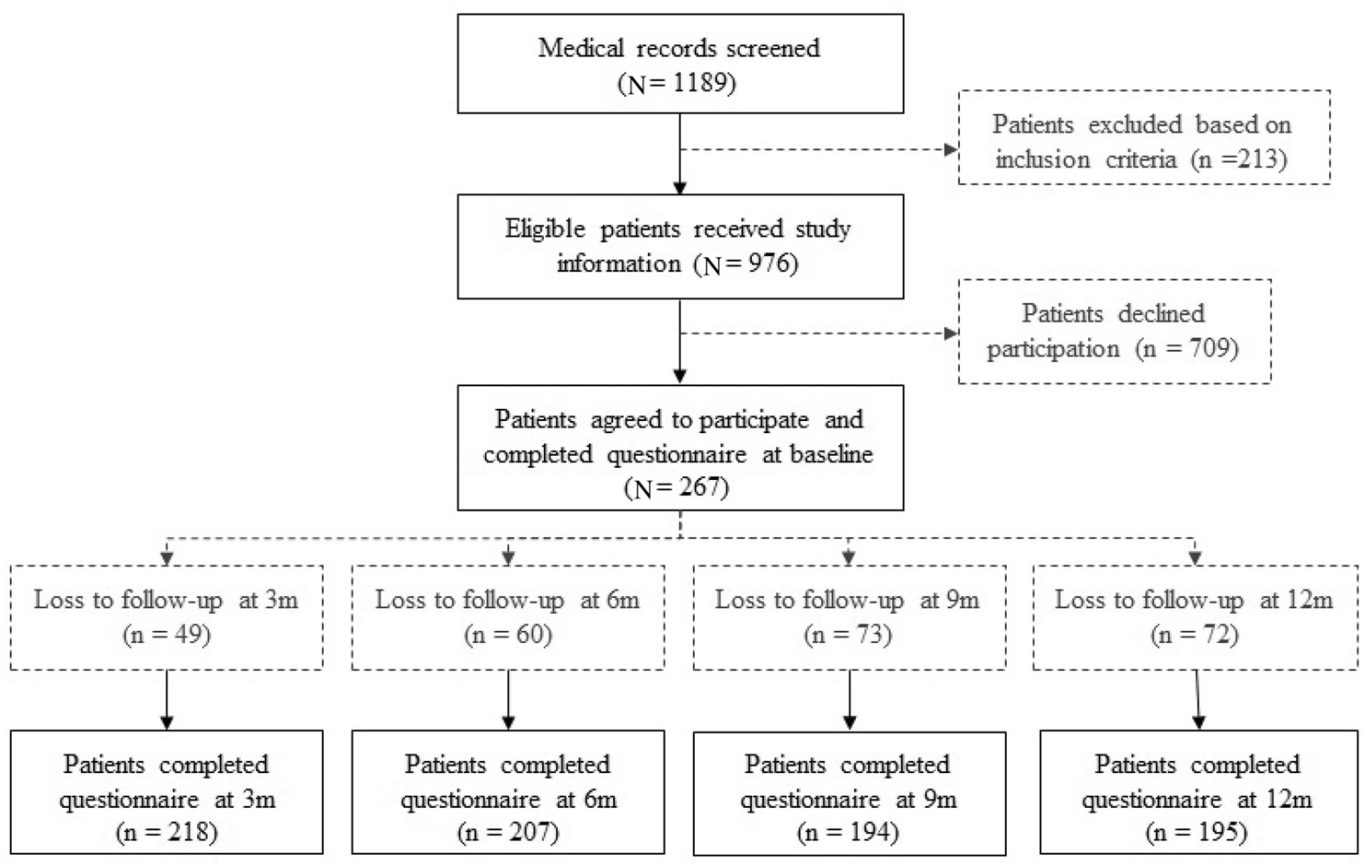
Flowchart of study population

In total, 267 patients were included at baseline (27% response rate, see Fig. 1). The response rate at three, six, nine, and 12 months follow-up was 81.6%, 77.5%, 72.7%, and 73.0%, respectively. The mean age of participants was 54.0 (SD = 16.1) and 61.8% were male patients. Moreover, participants showed more spinal cord injuries, thorax or abdominal with a combination of other injuries, and multi-trauma or burn wounds than non-participants. With regard to the nature of the injury, participants experienced more often a trauma as cyclist and they more often had an isolated head injury compared to non-participants. Patients’ sociodemographic and medical aspects are shown in Table 1.

**Table 1:** Characteristics of the total cohort, participants who completed the baseline questionnaire, and non-participants who have been excluded from analysis

	Total cohort (*N* = 973)	Participants (*N* = 267)	Non-participants (*N* = 706)	*p*-value
Age (years)*	50.7 ± 20.0	54.0 ± 16.1	49.5 ± 21.2	** < .001**
18–44^‡^	358 (36.8)	61 (22.8)	297 (42.1)	
45–64^‡^	353 (36.3)	133 (49.8)	220 (31.2)	
65–74^‡^	131 (13.5)	52 (19.5)	79 (11.2)	
≥ 75^‡^	131 (13.5)	21 (7.9)	110 (15.6)	
Sex				.882
Female	368 (37.8)	102 (38.2)	266 (37.7)	
Male	605 (62.2)	165 (61.8)	440 (62.3)	
Trauma mechanism				**.014**
Motor vehicle accident	217 (22.3)	61 (22.8)	156 (22.1)	
Motorcycle	98 (10.1)	31 (11.6)	67 (9.5)	
Pedal cycle^‡^	185 (19.0)	64 (24.0)	121 (17.1)	
Pedestrian	20 (2.1)	4 (1.5)	16 (2.3)	
Fall	364 (37.4)	92 (34.4)	272 (38.6)	
Struck by/collision	66 (6.8)	15 (5.6)	51 (7.2)	
Other^‡^	23 (2.4)	0 (0)	23 (3.3)	
Number of injuries*	2.0 [0.0–31.0]	3.0 [2.0–7.0]	2.0 [0.0–11.0]	** < .001**
0–2^‡^	591 (60.7)	116 (43.4)	475 (67.3)	
3–5^‡^	301 (30.9)	107 (40.1)	194 (27.5)	
6–8^‡^	53 (5.4)	23 (8.6)	30 (4.2)	
≥ 9^‡^	28 (2.9)	21 (7.9)	7 (1.0)	
Type/nature of injury				** < .001**
Isolated head injury^‡^	71 (7.3)	7 (2.6)	64 (9.1)	
Head and other injuries	351 (36.1)	93 (34.8)	258 (36.5)	
Spinal cord injury	100 (10.3)	30 (11.2)	70 (9.9)	
Orthopedic injuries only	131 (13.5)	27 (10.1)	104 (14.7)	
Chest/abdominal alone	51 (5.2)	12 (4.5)	39 (5.5)	
Chest/abdominal and other injuries	66 (6.8)	24 (9.0)	42 (5.9)	
Other multi-trauma and burn^‡^	191 (19.6)	74 (27.7)	117 (16.6)	
Other^‡^	10 (1.0)	0 (0)	10 (1.4)	
ISS score*^ǂ^	*N* = 609	*N* = 263	*N* = 346	** < .001**
	5.0 [1.0–48.0]	5.0 [2.0–9.0]	6.0 [1.0–48.0]	
1–3	209 (34.3)	111 (42.2)	98 (28.3)	
4–8	157 (25.8)	71 (27.0)	86 (24.9)	
9–15	120 (19.7)	47 (17.9)	73 (21.1)	
≥ 16	123 (20.2)	34 (12.9)	89 (25.7)	
Glasgow Coma Score*	14.6 ± 1.0	14.7 ± 0.8	14.6 ± 1.1	.156
9–12	45 (4.7)	8 (3.0)	37 (5.2)	
13–15	914 (95.3)	259 (97.0)	655 (92.8)	
Hospitalization				** < .001**
Yes	519 (53.3)	173 (64.8)	346 (49.0)	
No	454 (46.7)	94 (54.3)	360 (51.0)	
Admission to ICU^ǂ^				** < .001**
Yes	138 (26.6)	36 (20.8)	102 (29.5)	
No	381 (73.4)	137 (79.2)	244 (70.5)	
Length of stay*	7.2 [0.0–124.0]	3.0 [0.0–29.0]	8.3 [1.0–124.0]	**.010**
1–2 days	204 (21.0)	76 (28.5)	128 (18.1)	
3–7 days	165 (17.0)	54 (20.2)	111 (15.7)	
8–14 days	77 (7.9)	21 (7.9)	56 (7.9)	
> 15 days	60 (6.2)	9 (3.4)	51 (7.2)	
Surgery		43 (25.1)		
Living situation				
Alone		45 (16.9)		
With parents		18 (6.7)		
With a partner, no children		101 (37.8)		
With a partner and children		86 (32.2)		
Alone, with children		15 (5.6)		
Educational level				
Low		49 (19.7)		
Middle		103 (41.4)		
High		97 (39.0)		
Employment				
Employed		159 (59.8)		
Unemployed		108 (40.2)		
Psychiatric history^¥^		17 (6.4)		
Treatment by health psychologist after trauma		4 (1.5)		

*ICU* Intensive Care Unit, *ISS* Injury severity score

Bold indicates the *p*-value less than .05 (*p* ≤ .05) is statistically significant

^*^Means ± standard deviations or the median [Min–Max]. Number of patients (percentages) are provided for categorical variables. Missing data were not included in calculating percentages

^‡^A significant difference between the participants and non-participants

^ǂ^Admission to the ICU could be calculated only for patients who were hospitalized after treatment in the shock room and not for patients who were discharged after treatment in the shock room. ISS scores could, in the majority of cases, be calculated, especially in hospitalized patients

The missing sum scores for QOL are presented in Supplemental Table 1. Concerning the IES-R, 21 (7.9%) missing item scores were imputed, whereas 3 (1.1%) missing item scores for the HADS anxiety and 1 (0.4%) missing item score for HADS depression were imputed.

Table 2 indicates that four similar latent trajectory classes best fitted the data for psychological health and environment, based on the lowest BIC value criterion. Five different trajectories best fitted the data for physical health and social relationships. Seven trajectories were found for overall QOL and general health. The labels of the trajectories were based on total mean scores on each domain at baseline, when they seemed to be stable during 12 months after trauma. Otherwise, in case of change in direction, the labels of the trajectories were based on the course of QOL scores across time (e.g., Recovery) and compared with norm scores [29]. Tables 3, 4, 5, and 6 show the sociodemographic, clinical, and psychological characteristics of patients classified in each trajectory. Table 6 shows for each QOL domain the characteristics that most strongly predict the difference between the highest and lowest scoring QOL trajectories over the 12-month follow-up.

**Table 2:** The number of parameters and the log-likelihood were used to calculate the Bayesian information criterion (BIC) values of all models for quality of life domains over 12 months

N. of classes	NPar	Physical health		Psychological health		Social relationships		Environment		Overall QOL and general health	
		LL	BIC	LL	BIC	LL	BIC	LL	BIC	LL	BIC
1	6	− 2789.3	5612.2	− 2631.2	5295.9	− 2691.3	5416.1	− 2525.6	5084.6	− 2132.7	4298.9
2	13	− 2521.2	5115.0	− 2316.4	4705.5	− 2448.4	4969.4	− 2237.6	4547.8	− 1920.5	3913.6
3	20	− 2450.2	5012.2	− 2222.1	4555.9	− 2391.6	4894.9	− 2085.0	4281.6	− 1830.6	3773.0
4	27	− 2415.5	4981.8	− **2153.8**	**4458.5**	− 2348.3	4847.5	− **2033.7**	**4218.2**	− 1791.9	3734.6
5	34	− **2395.9**	**4981.7**	− 2134.4	4458.8	− **2325.0**	**4839.9**	− 2014.4	4218.7	− 1711.6	3613.1
6	41	− 2377.1	4983.3	− 2121.1	4471.2	− 2310.4	4849.8	− 2002.2	4233.5	− 1690.9	3610.9
7	48	− 2365.2	4998.5	− 2111.9	4491.9	− 2301.5	4871.2	− 1993.5	4255.1	− **1669.0**	**3606.2**
8	55	− 2352.3	5011.9	− 2106.1	4519.4	− 2280.5	4868.4	− 1981.6	4270.4	− 1651.1	3609.6
9	62	− 2339.1	5024.7	− 2094.0	4534.3	− 2271.2	4888.9	− 1973.9	4294.3	− 1629.9	3606.3
10	69	− 2325.2	5035.9	− 2086.7	4558.9	− 2261.0	4907.5	− 1972.1	4329.8	− 1618.9	3623.4

The BIC value for the final model is marked in bold.

*QOL* quality of life, *N* number, *NPar* number of parameters, *LL* log-likelihood, *BIC* Bayesian Information Criterion

The optimum number of classes is based on the BIC. This is an indicator for model fit (LL) and it takes complexity of de model with number of parameters (NPar) into account. The number of parameters is the same for each class

### Trajectories for physical health

**Fig. 2:**
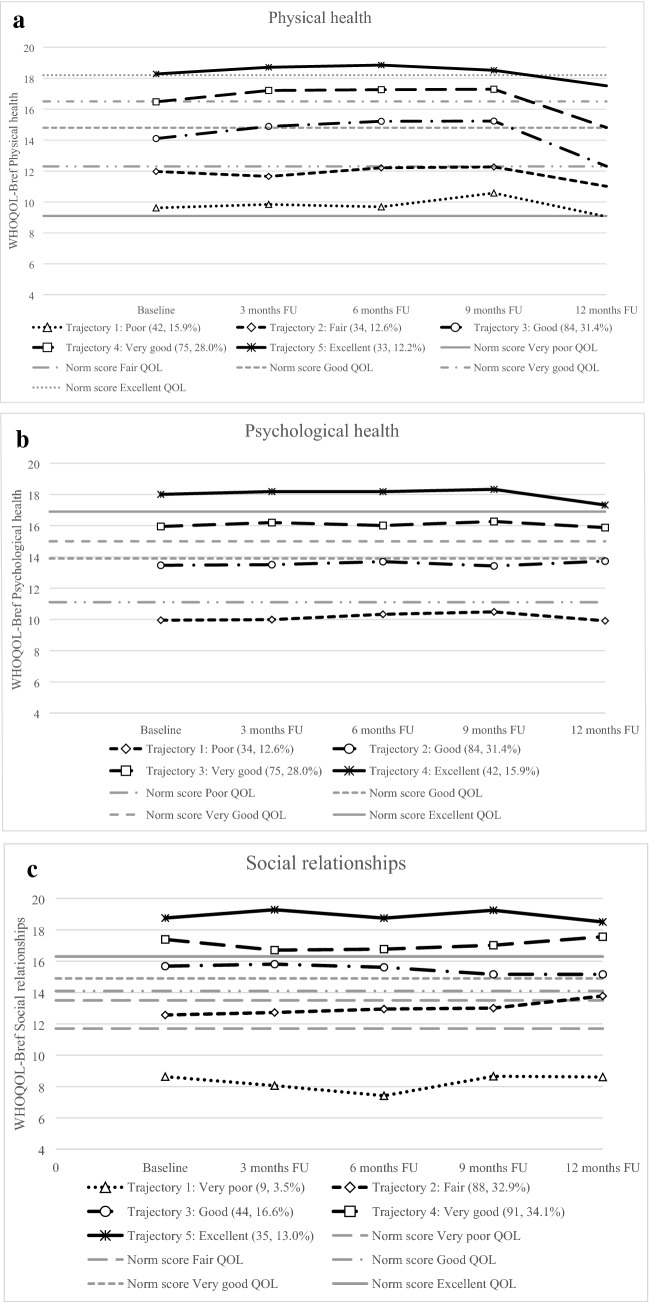

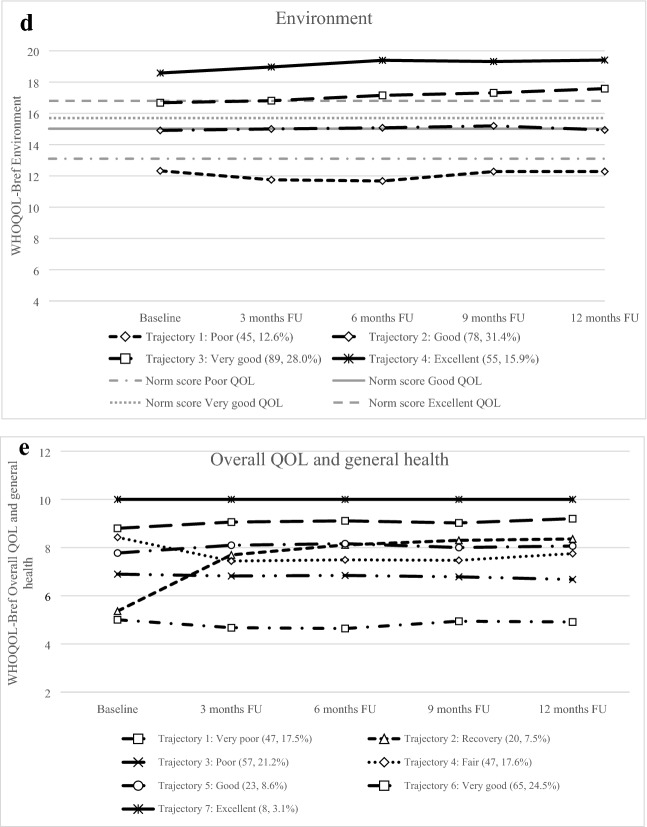
a. Trajectories of physical health. *WHOQOL-Bref* World Health Organization Quality of Life assessment instrument-Bref. Notes: Class means are shown. A higher score indicates a better quality of life. Number of patients and percentages are shown of the sample included in each class. Norm scores are provided for Very poor QOL, Fair QOL, Good QOL, Very good QOL, and Excellent QOL. **b.** Trajectories of Psychological health. *WHOQOL-Bref* World Health Organization Quality of Life assessment instrument-Bref. Notes: Class means are shown. A higher score indicates a better quality of life. Number of patients and percentages are shown of the sample included in each class. Norm scores are provided for Poor QOL, Good QOL, Very good QOL, and Excellent QOL **c.** Trajectories of Social relationships. *WHOQOL-Bref* World Health Organization Quality of Life assessment instrument-Bref. Notes: Class means are shown. A higher score indicates a better quality of life. Number of patients and percentages are shown of the sample included in each class. Norm scores are provided for Very poor QOL, Fair QOL, Good QOL, Very good QOL, and Excellent QOL. **d.** Trajectories of Environment. *WHOQOL-Bref* World Health Organization Quality of Life assessment instrument-Bref. *Notes*: Class means are shown. A higher score indicates a better quality of life. Number of patients and percentages are shown of the sample included in each class. Norm scores are provided for Poor QOL, Good QOL, Very good QOL, and Excellent QOL. **e.** Trajectories of Overall QOL and general health. *WHOQOL-Bref* World Health Organization Quality of Life assessment instrument-Bref, QOL: quality of life. Notes: Class means are shown. A higher score indicates a better quality of life. Number of patients and percentages are shown of the sample included in each class

The five trajectories were labeled as Poor, Fair, Good, Very good, and Excellent (see Table 3 and Fig. 2a). The identified physical health trajectories differed significantly on all investigated psychological characteristics, except for Agreeableness and Openness. Patients in both the Poor and Fair class scored significantly more often on ASD (*p* = 0.002) and higher on anxiety, depressive symptoms, PTSD, Neuroticism, trait anxiety, and lower on Extraversion and Conscientiousness compared with the other three trajectories (i.e., Good, Very good, and Excellent). No significant differences were found for sociodemographic and clinical characteristics.

**Table 3:** Sociodemographic, clinical, and psychological characteristics for the five trajectories of physical health and social relationships

Characteristics	Physical health					
	Trajectory 1: Poor	Trajectory 2: Fair	Trajectory 3: Good	Trajectory 4: Very good	Trajectory 5: Excellent	p-value
	42 (15.9)	34 (12.6)	84 (31.4)	75 (28.0)	33 (12.2)	
Anxiety*	9.2 ± 3.7^4,5^	10.6 ± 3.2^3,4,5^	6.9 ± 4.8^2,5^	5.4 ± 4.3^1,2^	3.8 ± 4.4^1,2,3^	** < .001**
Depressive symptoms*	6.9 ± 2.7^3,4,5^	6.9 ± 2.5^3,4,5^	5.0 ± 2.6^1,2^	4.3 ± 2.1^1,2^	4.2 ± 2.3^1,2^	** < .001**
Neuroticism*	34.2 ± 8.6^3,4,5^	34.9 ± 8.2^3,4,5^	28.1 ± 7.2^1,2^	26.4 ± 6.5^1,2^	24.8 ± 6.8^1,2^	** < .001**
Trait anxiety*	21.9 ± 7.8^3,4,5^	22.0 ± 5.9^3,4,5^	17.0 ± 5.1^1,2^	14.8 ± 3.8^1,2^	14.0 ± 3.4^1,2^	** < .001**
PTSD*	34.7 ± 21.2^3,4,5^	26.0 ± 16.8^3,4,5^	15.6 ± 14.3^1,2^	10.8 ± 11.7^1,2^	10.2 ± 12.9^1,2^	** < .001**
Extraversion*	38.5 ± 7.1^3,4,5^	38.4 ± 5.9^3,4,5^	42.5 ± 5.3^1,2^	43.6 ± 6.4^1,2^	42.8 ± 7.2^1,2^	** < .001**
Conscientiousness*	44.1 ± 7.5	41.5 ± 6.2^3,4,5^	45.6 ± 5.8^2^	46.5 ± 5.4^2^	47.4 ± 6.0^2^	** < .001**
ASD (yes)	9 (22.5)^‡^	3 (10.0)	4 (5.4)	2 (2.6)	0 (0)	.**001**
GCS*	14.8 ± 0.6	14.3 ± 1.5	14.9 ± 0.4	14.7 ± 0.8	14.6 ± 0.9	.022
Hospital stay on the ICU (yes)	4 (16.0)	7 (35.0)	8 (15.1)	16 (31.4)	1 (4.2)	.023
Psychiatric history	7 (17.5)	1 (3.2)	5 (6.0)	2 (2.5)	2 (6.1)	.028
Agreeableness*	40.7 ± 4.5	40.1 ± 4.7	42.1 ± 4.9	42.5 ± 3.9	42.2 ± 4.3	.054
LOS*	5.6 ± 6.0	5.8 ± 6.0	4.5 ± 4.6	5.6 ± 6.7	1.8 ± 1.3	.057
Age*	50.5 ± 16.8	49.5 ± 14.7	56.3 ± 16.2	55.9 ± 15.3	52.5 ± 15.1	.117
Living together (yes)	27 (69.2)	25 (80.6)	71 (86.6)	69 (86.3)	28 (84.8)	.147
Sex (male)	20 (50.0)	16 (51.6)	51 (61.4)	57 (71.3)	21 (63.6)	.147
Paid job (yes)	20 (51.3)	17 (54.8)	50 (60.2)	46 (57.5)	26 (78.8)	.157
Education (high)	12 (32.4)	9 (29.0)	24 (31.6)	34 (46.6)	18 (56.3)	.176
Surgery (yes)	9 (37.5)	7 (35.0)	13 (24.5)	11 (21.6)	3 (13.0)	.275
ISS*	6.8 ± 6.9	8.7 ± 10.0	6.9 ± 7.1	6.9 ± 6.2	4.9 ± 5.2	.327
Hospital stay (yes)	25 (62.5)	20 (64.5)	53 (63.9)	51 (63.8)	24 (72.7)	.898
Openness*	36.4 ± 7.8	36.8 ± 5.0	34.7 ± 6.3	35.0 ± 5.5	36.6 ± 6.6	.284

Characteristics	Social relationships					
	Trajectory 1: Very poor	Trajectory 2: Fair	Trajectory 3: Good	Trajectory 4: Very good	Trajectory 5: Excellent	*p*-value
	9 (3.5)	88 (32.9)	44 (16.6)	91 (34.1)	35 (13.0)	
Anxiety*	12.3 ± 2.3^3,4,5^	8.8 ± 4.5^3,4,5^	6.3 ± 4.4^1,2^	5.4 ± 4.4^1,2^	4.8 ± 4.4^1,2^	** < .001**
Depressive symptoms*	8.1 ± 2.4^3,^^4,5^	5.8 ± 2.6^4,5^	5.4 ± 2.6^1,5^	4.6 ± 2.4^1,2^	3.3 ± 2.0^1,2,3^	** < .001**
Neuroticism*	40.4 ± 8.0^2,3,4,5^	32.3 ± 7.3^1,3,4,5^	27.7 ± 6.0^1,2^	26.7 ± 7.8^1,2^	23.1 ± 6.6^1,2^	** < .001**
Trait anxiety*	28.3 ± 5.2^2,3,4,5^	10.1 ± 6.3^1,3,4,5^	16.2 ± 4.4^1,2^	16.0 ± 5.3^1,2^	13.1 ± 3.3^1,2^	** < .001**
PTSD*	36.7 ± 21.4^3,4,5^	24.4 ± 18.7^3,4,5^	13.0 ± 12.8^1,2^	13.4 ± 15.1^1,2^	11.2 ± 11.5^1,2^	** < .001**
Extraversion*	33.6 ± 8.7^3,4,5^	39.8 ± 5.9^4,5^	42.6 ± 5.3^1^	43.2 ± 6.3^1,2^	44.7 ± 7.5^1,2^	** < .001**
Conscientiousness*	38.7 ± 10.5^4,5^	44.1 ± 6.4^5^	44.8 ± 4.5	46.9 ± 6.0^1,2^	47.2 ± 4.9^1^	** < .001**
ASD	3 (37.5)^‡^	11 (12.9)^‡^	1 (2.6)	3 (3.0)^‡^	0 (0)	** < .001**
Agreeableness*	40.9 ± 4.6	40.4 ± 4.1	42.2 ± 4.0	42.8 ± 4.6	41.6 ± 5.4	.005
Education (high)	3 (37.5)	23 (26.7)	16 (38.1)	47 (49.5)	8 (44.4)	.053
Age*	45.3 ± 16.7	52.1 ± 15.3	59.5 ± 16.2	54.3 ± 16.4	52.7 ± 15.0	.063
Psychiatric history	2 (25.0)	8 (9.0)	3 (6.8)	3 (2.8)	1 (5.6)	.090
Sex (male)	4 (50.0)	62 (69.7)	30 (68.2)	58 (53.7)	11 (61.1)	.161
ISS*	2.9 ± 2.8	6.9 ± 7.5	8.1 ± 7.8	6.3 ± 6.4	8.6 ± 6.7	.231
Living together (yes)	6 (75.0)	69 (79.3)	41 (93.2)	88 (81.5)	16 (88.9)	.281
Hospital stay (yes)	4 (50.0)	55 (61.8)	33 (75.0)	67 (62.0)	14 (77.8)	.307
Openness*	37.9 ± 4.8	34.7 ± 5.9	36.3 ± 6.5	35.5 ± 6.4	37.2 ± 6.7	.342
Paid job (yes)	3 (37.5)	56 (63.6)	22 (50.0)	66 (61.1)	12 (66.7)	.357
Surgery (yes)	0 (0)	12 (21.8)	11 (33.3)	16 (24.6)	4 (28.6)	.568
GCS*	14.9 ± 0.4	14.7 ± 0.9	14.8 ± 0.4	14.7 ± 0.8	14.6 ± 1.2	.716
LOS*	1.7 ± 1.2	4.9 ± 5.6	4.7 ± 4.5	4.6 ± 5.6	5.8 ± 7.4	.824
Hospital stay on the ICU (yes)	0 (0)	13 (23.6)	7 (21.2)	13 (19.4)	3 (21.4)	.843

Number of patients (percentages) are provided for categorical variables

*PTSD* post-traumatic stress disorder, *ASD* acute stress disorder, *LOS* length of stay, *ISS* injury severity score, *GCS* Glasgow Coma Score, *ICU* intensive care unit

*Means ± standard deviations. Missing data were not included in calculating percentages

^‡^^,1,2,3,4,5^A significant difference between the specified class(es). Using a Holm adjusted significance level, significant *p*-values for differences in a characteristic between all classes are shown in bold. Ranking of characteristics is based on *p*-value (low–high)

The most pronounced differences between the Excellent trajectory and Poor trajectory were found for PTSD, trait anxiety, and anxiety. Patients with Poor physical health trajectories had substantially higher baseline scores on PTSD, trait anxiety, and anxiety than patients with Excellent physical health. Patients in the Poor physical health trajectory significantly more often had ASD at baseline than patients with Excellent physical health trajectories (*n* = 9, 22.5% versus *n* = 0, 0%, *r*_*φ*_ = 0.27, *p* = 0.024).

### Trajectories for psychological health

The four identified trajectories were labeled as Poor, Good, Very good, and Excellent psychological health (see Fig. 2b). These trajectories differed significantly on all examined psychological factors, except for Agreeableness and Openness (see Table 4). Sociodemographic and clinical factors did not significantly differ between the psychological health trajectories. Patients with Poor psychological health scored more often on ASD (*p* < 0.001) and higher on anxiety, depressive symptoms, PTSD, Neuroticism, trait anxiety, and lower on Extraversion and Conscientiousness compared to the other three trajectories (i.e., Good, Very good, and Excellent). Patients in the Very good psychological health trajectory showed significantly less ASD symptoms compared with other trajectories (i.e., Poor and Good).

**Table 4:** Sociodemographic, clinical, and psychological characteristics for the four trajectories of psychological health and environment

Characteristics	Psychological health				
	Trajectory 1: Poor	Trajectory 2: Good	Trajectory 3: Very good	Trajectory 4: Excellent	*p*-value
	34 (12.6)	84 (31.3)	75 (28.0)	42 (15.6)	
Anxiety*	11.3 ± 2.5^2,3,4^	9.0 ± 4.2^1,3,4^	4.7 ± 3.8^1,2^	3.9 ± 4.0^1,2^	< .001
Depressive symptoms*	8.1 ± 2.1^2,3,4^	5.7 ± 2.4^1,3,4^	4.0 ± 2.1^1,2^	4.2 ± 2.3^1,2^	** < .001**
Neuroticism*	39.1 ± 6.3^2,3,4^	31.3 ± 6.4^1,3,4^	25.8 ± 6.5^1,2^	23.4 ± 5.9^1,2^	** < .001**
Trait anxiety*	26.4 ± 6.5^2,3,4^	18.7 ± 4.6^1,3,4^	14.6 ± 3.1^1,2^	13.2 ± 2.9^1,2^	** < .001**
PTSD*	37.6 ± 21.2^2,3,4^	20.4 ± 14.9^1,3,4^	9.9 ± 11.5^1,2,4^	11.7 ± 13.7^1,2^	** < .001**
Extraversion*	36.0 ± 6.0^2,3,4^	41.4 ± 5.9^1,4^	42.3 ± 5.9^1^	45.0 ± 6.3^1,2^	** < .001**
Conscientiousness*	41.6 ± 7.8^3,4^	44.3 ± 6.1^4^	46.0 ± 5.3^1^	48.3 ± 5.0^1,2^	** < .001**
ASD (yes)	10 (28.6)^‡^	7 (7.9)	0 (0)^‡^	1 (1.8)	** < .001**
Agreeableness*	39.5 ± 4.7	41.6 ± 4.1	42.1 ± 4.5	42.9 ± 4.6	.004
Psychiatric history	21 (60.0)	53 (57.6)	51 (65.4)	40 (64.5)	.005
Age*	47.6 ± 16.4	52.0 ± 16.4	57.4 ± 15.4	56.5 ± 15.2	.008
Education (high)	8 (23.5)	29 (33.3)	33 (46.5)	27 (47.4)	.010
Living together (yes)	23 (65.7)	76 (84.4)	68 (87.2)	53 (58.8)	.032
Paid job (yes)	17 (48.6)	59 (64.8)	46 (59.0)	37 (59.7)	.421
GCS*	14.9 ± 0.6	14.6 ± 1.0	14.7 ± 0.8	14.8 ± 0.8	.455
ISS*	5.5 ± 6.5	6.9 ± 7.7	6.7 ± 6.6	7.7 ± 6.7	.521
Sex (male)	21 (60)	53 (57.6)	51 (65.4)	40 (64.5)	.717
LOS*	4.5 ± 4.9	5.2 ± 5.4	5.0 ± 6.3	4.0 ± 5.0	.743
Hospital stay (yes)	21 (60.0)	58 (63.0)	51 (65.4)	43 (69.4)	.788
Openness*	35.1 ± 5.5	35.9 ± 6.8	35.3 ± 6.7	35.5 ± 5.2	.904
Surgery (yes)	4 (20.0)	15 (25.9)	13 (26.0)	11 (25.6)	.956
Hospital stay on the ICU (yes)	4 (19.0)	13 (22.4)	10 (19.6)	9 (20.9)	.981

Characteristics	Environment				
	Trajectory 1: Poor	Trajectory 2: Good	Trajectory 3: Very good	Trajectory 4: Excellent	*p*-value
	45 (16.8)	78 (29.0)	89 (33.4)	55 (20.7)	
Anxiety*	9.7 ± 4.0^3,4^	7.9 ± 4.4^3,4^	6.0 ± 4.6^1,2^	4.3 ± 4.4^1,2^	** < .001**
Depressive symptoms*	7.3 ± 2.5^2,3,4^	5.4 ± 2.5^1,3,4^	4.4 ± 2.4^1,2^	4.2 ± 2.0^1,2^	** < .001**
Neuroticism*	37.3 ± 7.4^,2,3,4^	29.3 ± 7.0^1,4^	26.8 ± 6.8^1^	24.8 ± 6.6 ^1,2^	** < .001**
Trait anxiety*	24.3 ± 6.9^2,3,4^	17.4 ± 5.1^1^	15.7 ± 4.3^1^	13.7 ± 3.1^1^	** < .001**
PTSD (IES-R)*	33.5 ± 20.9^2,3,4^	19.0 ± 15.7^1,3,4^	12.3 ± 12.2^1,2^	10.6 ± 13.5^1,2^	** < .001**
Extraversion*	37.9 ± 5.7^2,3,4^	41.9 ± 6.2^1^	42.4 ± 6.1^1^	43.8 ± 7.0^1^	** < .001**
Conscientiousness*	42.2 ± 7.4^2,3,4^	45.3 ± 6.3^1^	45.9 ± 5.1^1^	47.3 ± 5.8^1^	** < .001**
ASD (yes)	11 (24.4)^‡^	5 (6.9)	1 (1.3)^‡^	1 (1.9)	** < .001**
Agreeableness*	38.6 ± 3.8^2,3,4^	41.8 ± 4.2^1^	42.9 ± 4.3^1^	42.6 ± 4.7^1^	** < .001**
Education (high)	6 (13.6)^‡^	18 (25.4)^‡^	39 (50.0)^‡^	34 (60.7)^‡^	** < .001**
Psychiatric history	8 (17.4)	3 (3.8)	5 (5.9)	1 (1.7)	.006
Age*	47.5 ± 16.3	54.2 ± 16.7	55.7 ± 16.6	56.6 ± 13.3	.017
ISS*	4.6 ± 6.9	7.1 ± 7.7	6.8 ± 6.4	8.3 ± 6.8	.065
Paid job (yes)	21 (46.7)	43 (55.1)	55 (64.7)	40 (69.0)	.078
Openness*	35.1 ± 6.4	34.3 ± 6.5	35.9 ± 6.4	37.0 ± 5.4	.078
GCS*	14.9 ± 0.5	14.7 ± 0.7	14.6 ± 1.1	14.7 ± 0.8	.206
Hospital stay (yes)	24 (52.2)	50 (64.1)	59 (69.4)	40 (69.0)	.215
Living together (yes)	33 (73.3)	66 (85.7)	70 (82.4)	51 (87.9)	.220
ICU	3 (12.5)	14 (28.0)	10 (16.9)	9 (22.5)	.365
Sex (male)	26 (56.5)	52 (66.7)	50 (58.8)	37 (63.8)	.628
LOS*	4.5 ± 4.6	5.1 ± 5.4	5.1 ± 6.2	4.0 ± 5.1	.755
Surgery (yes)	5 (21.7)	14 (28.6)	16 (27.1)	8 (20.0)	.769

Number of patients (percentages) are provided for categorical variables

*PTSD* post-traumatic stress disorder, *ASD* acute stress disorder, *LOS* length of stay, *ISS* injury severity score, *GCS* Glasgow Coma Score, *ICU* intensive care unit

*Means ± standard deviations. Missing data were not included in calculating percentages

^‡,1,2,3,4^A significant difference between the specified class(es). Using a Holm adjusted significance level, significant *p*-values for differences in a characteristic between all classes are shown in bold. Ranking of characteristics is based on *p*-value (low–high)

The most pronounced differences between the Excellent (class 4; reference group) trajectory and Poor psychological health trajectory were found for trait anxiety, Neuroticism, and anxiety. Patients with Poor psychological health had substantially higher baseline scores on trait anxiety, Neuroticism, and anxiety than patients with Excellent psychological health. Patients in the Poor psychological health trajectory more often had ASD at baseline than patients with patients with Excellent psychological health (*n* = 10, 28.6% versus *n* = 1, 1.8%, *r*_*φ*_ = 0.31, *p* = 0.007).

### Trajectories for social relationships

The five identified trajectories were labeled as Very poor, Fair, Good, Very good, and Excellent social relationships (see Table 3 and Fig. 2c). These trajectories differed significantly on all investigated psychological characteristics, except for Agreeableness and Openness. The trajectories did not differ in terms of sociodemographic and clinical characteristics. Patient in the Very poor and Fair social relationships trajectory scored more often on ASD (*p* < 0.001) and significantly higher on anxiety, depressive symptoms, PTSD, Neuroticism, and trait anxiety, and lower on Extraversion and Conscientiousness compared to the other three (i.e., Good, Very good, and Excellent) trajectories.

The most pronounced differences between the Excellent and Very poor social relationships trajectories were found for trait anxiety, Neuroticism, and depressive symptoms. Patients with Very poor trajectories scored substantially higher on trait anxiety, Neuroticism, and depressive symptoms than patients with Excellent trajectories. Patients with Very poor social relationships trajectories had more often ASD than patients with patients with Excellent social relationships (*n* = 3, 37.5% versus *n* = 0, 0%, *r*_*φ*_ = 0.45, *p* = 0.014).

### Trajectories for environment

The four identified trajectories were labeled as Poor, Good, Very good, and Excellent environmental QOL (see Table 4 and Fig. 2d). These trajectories differed significantly on all investigated psychological factors, except for Openness. The trajectories did not differ significantly in terms of clinical characteristics. Patients in the Poor environment trajectory scored significantly more often on ASD (*p* < 0.001) and higher on anxiety, depressive symptoms, PTSD, Neuroticism, and trait anxiety, and lower on Extraversion, Conscientiousness, and Agreeableness compared with the other (i.e., Good, Very good, and Excellent) trajectories.

The most pronounced differences between the Excellent trajectory and Poor trajectory were found for trait anxiety, Neuroticism, and depressive symptoms. Patients in the Poor trajectory scored at baseline substantially higher on trait anxiety, Neuroticism, and depressive symptoms than patients in the Excellent environment trajectory. Patients in the Poor environment trajectory had more often ASD at baseline (*n* = 11, 24.4%) than patients in the Excellent trajectory (*n* = 1, 1.9%, *r*_*φ*_ = 0.29, *p* = 0.006). More patients in the Excellent environment trajectory were higher educated (*n* = 34, 60.7%) compared to patients in the Poor trajectory (*n* = 6, 13.6%, *r*_*φ*_ = *− *0.28, *p* = 0.002).

### Trajectories for overall quality of life and general health

The seven identified trajectories were labeled as Very poor, Recovery, Poor, Fair, Good, Very good, and Excellent class (see Fig. 2e). These trajectories differed significantly on all investigated psychological factors, except for Conscientiousness, Agreeableness, and Openness (see Table 5). The trajectories did not significantly differ on the sociodemographic and clinical variables. Patients in the Very poor trajectory scored significantly higher on anxiety, depressive symptoms, PTSD, Neuroticism, and trait anxiety, and lower on Extraversion than patients in the other trajectories. Significantly more patients with ASD (*p* < 0.001) were found in the Very poor (trajectory 1, *n* = 13, 27.1%) trajectory compared with other trajectories, whereas no patients with ASD were found in the Very good (trajectory 6, *n* = 0, 0%) trajectory. The Recovery trajectory was the only trajectory in which QOL improved over time, from Very poor QOL at baseline to Good QOL at 12 months after trauma. These patients scored significantly higher on Extraversion and had significantly lower PTSD, Neuroticism, trait anxiety, and depression scores at baseline than patients who did not recover during the 12 months follow-up (i.e., Very Poor trajectory). Furthermore, patients in the Recovery trajectory were more often female patients with high education and longer hospital stay, though these results were not statistically significant.

**Table 5:** Sociodemographic, clinical, and psychological characteristics for the seven trajectories of overall quality of life and general health

Characteristics	Overall quality of life and general health							
	Trajectory 1: Very poor	Trajectory 2: Recovery	Trajectory 3: Poor	Trajectory 4: Fair	Trajectory 5: Good	Trajectory 6: Very good	Trajectory 7: Excellent	*p*-value
	47 (17.5)	20 (7.5)	57 (21.1)	47 (17.6)	23 (8.6)	65 (24.4)	8 (3.1)	
Anxiety*	9.6 ± 3.5^4,5,6,7^	7.6 ± 3.6^6^	9.0 ± 4.6^5,6,7^	6.5 ± 5.0^1,6^	5.8 ± 3.8^1,3^	3.7 ± 3.9^1,2,3,4^	3.9 ± 3.8^1,3^	** < .001**
Depressive symptoms*	7.6 ± 2.3^2,3,4,5,6,7^	5.4 ± 2.3^1^	5.2 ± 2.4^1^	4.7 ± 2.7^1^	4.3 ± 2.1^1^	4.1 ± 2.1^1^	4.3 ± 2.0^1^	** < .001**
Neuroticism*	35.8 ± 7.9^2,3,4,5,6,7^	28.1 ± 7.1^1^	31.3 ± 8.0^1,4,6^	26.2 ± 6.5^1,3^	27.4 ± 4.9^1^	24.7 ± 6.7^1,3^	25.4 ± 7.4^1^	** < .001**
Trait anxiety*	23.4 ± 7.5^2,3,4,5,6,7^	17.5 ± 5.2^1^	19.0 ± 4.7^1,4,5,6,7^	14.6 ± 3.9^1,3^	14.1 ± 2.5^1,3^	14.4 ± 4.0^1,3^	13.6 ± 3.7^1,3^	** < .001**
PTSD*	33.3 ± 21.8^2,3,4,5,6,7^	14.4 ± 14.0^1^	20.2 ± 14.9^1,5,6^	15.7 ± 14.2^1^	8.3 ± 11.3^1,3^	10.9 ± 11.8^1,3^	8.6 ± 12.1^1^	** < .001**
Extraversion*	38.1 ± 6.2^2,6^	44.6 ± 6.0^1^	40.8 ± 5.9	42.1 ± 6.7	42.6 ± 5.0	43.8 ± 6.6^1^	43.7 ± 7.7	** < .001**
ASD (yes)	13 (27.1)^‡^	1 (5.6)	1 (1.9)	2 (5.6)	1 (3.8)	0 (0)^‡^	0 (0)	** < .001**
Conscientiousness*	43.0 ± 7.2	46.4 ± 6.6	44.2 ± 5.6	45.1 ± 6.7	47.5 ± 3.9	47.0 ± 5.4	46.9 ± 8.1	.004
Psychiatric history	9 (18.8)	1 (5.6)	4 (6.8)	2 (5.3)	0 (0)	1 (1.5)	0 (0)	.007
Education (high)	11 (24.4)	11 (68.8)	14 (24.1)	13 (39.4)	11 (40.7)	32 (52.5)	5 (55.6)	.013
Sex (male)	26 (54.2)	6 (33.3)	37 (62.7)	26 (68.4)	23 (82.1)	43 (64.2)	4 (44.4)	.026
LOS*	5.3 ± 5.3	8.7 ± 6.8	5.6 ± 7.2	3.9 ± 3.1	5.6 ± 7.7	3.0 ± 2.8	2.3 ± 1.5	.035
Age*	50.7 ± 17.2	50.7 ± 13.2	53.1 ± 16.1	60.3 ± 11.5	54.5 ± 17.1	54.7 ± 17.9	52.3 ± 10.8	.176
Agreeableness*	40.3 ± 4.3	41.4 ± 5.0	41.6 ± 5.0	42.1 ± 4.8	41.5 ± 3.5	42.8 ± 4.2	42.7 ± 3.6	.180
Paid job (yes)	22 (46.8)	14 (77.8)	36 (61.0)	21 (55.3)	19 (67.9)	40 (59.7)	7 (77.8)	.235
Hospital stay on the ICU (yes)	8 (25.8)	3 (25.0)	7 (20.0)	3 (10.7)	7 (38.9)	6 (14.0)	2 (33.3)	.260
Living together (yes)	34 (72.3)	16 (88.9)	49 (83.1)	31 (83.8)	26 (92.9)	56 (83.6)	8 (88.9)	.378
ISS*	6.8 ± 8.3	10.1 ± 7.8	6.3 ± 7.3	6.6 ± 5.5	7.5 ± 7.2	6.2 ± 6.0	7.4 ± 7.9	.541
GCS*	14.6 ± 1.0	14.4 ± 1.3	14.8 ± 0.8	14.8 ± 0.5	14.8 ± 0.5	14.7 ± 0.8	14.6 ± 0.7	.597
Surgery (yes)	7 (23.3)	4 (33.3)	8 (22.9)	8 (28.6)	6 (35.3)	10 (23.3)	0 (0)	.707
Openness*	34.6 ± 6.4	37.1 ± 8.5	35.2 ± 6.1	35.6 ± 5.7	34.8 ± 6.0	36.1 ± 6.0	36.8 ± 7.2	.744
Hospital stay (yes)	43 (64.2)	12 (66.7)	35 (59.3)	28 (73.7)	18 (64.3)	43 (64.2)	6 (66.7)	.906

Number of patients (percentages) are provided for categorical variables

*PTSD* post-traumatic stress disorder, *ASD* acute stress disorder, *LOS* length of stay, *ISS* injury severity score, *GCS* Glasgow Coma Score, *ICU* intensive care unit

*Means ± standard deviations. Missing data were not included in calculating percentages

^‡,1,2,3,4,5,6,7^ A significant difference between the specified class(es). Using a Holm adjusted significance level, significant *p*-values for differences in a characteristic between all classes are shown in bold. Ranking of characteristics is based on *p*-value (low–high)

**Table 6:** Cohens d effect sizes and Phi coefficients between Excellent and (Very) Poor QOL trajectories for all quality of life domains and overall QOL and general health

Characteristics	Physical health		Psychological health		Social relationships		Environment		Overall QOL and general health	
	Cohens’ d (Excellent vs. Poor)	CI interval (95%)	Cohen’s d (Excellent vs. Poor)	CI interval (95%)	Cohen’s d (Excellent vs. Very poor)	CI interval (95%)	Cohen’s d (Excellent vs. Poor)	CI interval (95%)	Cohen’s d (Excellent vs. Very poor)	CI interval (95%)
Anxiety	1.34	[.83, 1.85]	2.17	[1.60, 2.74]	1.84	[1.01, 2.66]	1.28	[.85, 1.71]	1.61	[.80, 2.42]
Depressive symptoms	1.07	[.58, 1.55]	1.76	[1.23, 2.29]	2.31	[1.43, 3.18]	1.38	[0.95, 1.82]	1.46	[.66, 2.26]
Neuroticism	1.20	[.70, 1.69]	2.58	[1.97, 3.19]	2.51	[1.61, 3.41]	1.79	[1.33, 2.26]	1.33	[0.54, 2.12]
Trait anxiety	1.26	[.76, 1.76]	2.72	[2.10, 3.35]	4.07	[2.95, 5.19]	2.05	[1.57, 2.54]	1.38	[.58, 2.17]
PTSD	1.36	[.85, 1.86]	1.48	[.97, 1.99]	1.83	[1.00, 2.66]	1.33	[0.89, 1.76]	1.19	[.41, 1.97]
Extraversion	− .60	[− 1.07, − .14]	− 1.46	[− 1.97, − .95]	− 1.43	[− 2.23, − .64]	− .91	[− 1.33, − .50]	− .87	[− 1.64, − .11]
Conscientiousness	− .48	[− .94, − .02]	− 1.05	[− 1.53, − .56]	− 1.34	[− 2.12, − .55]	− .78	[− 1.19, − .37]	–	–
Agreeableness	–	–	–	–	–	–	− .93	[− 1.34, − .51]	–	–
	*r*_*φ*_	*p*-value	*r*_*φ*_	*p*-value	*r*_*φ*_	*p*-value	*r*_*φ*_	*p*-value	*r*_*φ*_	*p*-value
ASD (yes)*	.27	.024	.31	.007	.45	.014	.29	.006	.18	.203
Education (high)*	–	–	–	–	–	–	− .28	.002	–	–

Excellent trajectory is the reference class

A positive Cohen’s d indicates a higher mean score for patients in the Poor or Very poor trajectory (class 1) compared to patients in the Excellent trajectory (class 4, 5, or 7; reference group), whereas a negative Cohen’s d indicates a lower mean score for patients in the Poor or Very poor trajectory (class 1) compared to patients in either the Excellent trajectory (class 4, 5, or 7; reference group). If the 95% confidence interval does not contain the null hypothesis value (zero), the results are statistically significant

*QOL* quality of life, *vs* versus, *CI* confidence interval, *ASD* acute stress disorder

*Phi coefficients are provided for ASD and education. -No significant differences were found for Conscientiousness in Overall QOL and general health, Agreeableness in Physical, Psychological, Social, and Overall QOL and general health, and education in Physical, Psychological, Social, and Overall QOL and general health

The most pronounced differences between the Excellent trajectory and Very poor trajectory were found for anxiety, depressive symptoms, and trait anxiety. Patients in the Very poor trajectory had substantially higher baseline scores on anxiety, depressive symptoms, and trait anxiety than patients in the Excellent trajectory.

## Discussion

To our knowledge, this is the first study that examined QOL trajectories and determined sociodemographic, clinical, and psychological characteristics of trajectory membership in physical trauma patients using a biopsychosocial approach. An overall finding is that psychological, but not sociodemographic or clinical aspects, defined trajectories. Furthermore, four latent trajectories were found for psychological health and environment, five for physical health and social relationships, and seven trajectories for overall QOL and general health. This study showed that patients at risk for impaired QOL can be identified at baseline based on symptoms of anxiety, depressive symptoms, acute stress disorder, post-traumatic stress disorder, Neuroticism, and trait anxiety and in general not on sociodemographic or clinical characteristics.

Although earlier research focused on improvement of HRQoL or HS [18, 21, 22, 50], the present study is the first to examine recovery on QOL domains. A Recovery trajectory was not found for the separate domains, but only for overall QOL and general health. At baseline, these patients had significantly less PTSD, depressive symptoms, Neuroticism, and trait anxiety than patients who did not improve their QOL during 12-months follow-up. Patients in the Recovery trajectory also showed significantly higher scores on Extraversion (at baseline) than patients in other trajectories. Finally, patients showing a Recovery trajectory more were often female patients, higher educated, and they had a longer hospital stay, than patients from other QOL trajectories. However, these results failed to reach statistical significance. Even though, these latter findings should be interpreted with caution, they may be interesting areas of future research.

Previous research identified psychological characteristics (e.g., anxiety, depressive symptoms, and PTSD) for impaired QOL [4, 9, 16], which were also relevant in this study. Compared to other trajectories, Very poor or Poor trajectories were characterized by ASD at baseline. This was also confirmed by the result that experiencing ASD symptoms is strongly related to impaired QOL [51]. A high score on the MINI-Plus does not necessarily mean that someone is diagnosed with ASD, because such a diagnosis requires the presence of symptoms on all domains (i.e., intrusion, negative emotions, dissociation, avoidance, and arousal). Therefore, ASD was used as a dichotomous variable. However, the other characteristics were used as continuous variables, because they indicate symptom severity. In addition, information about the relation between ASD on QOL is scarce, possibly because ASD is a relatively new diagnosis and less studied compared to PTSD [52]. Therefore, more research is needed that examines ASD in relation to QOL. Moreover, in line with previous studies, the association between personality traits and QOL was confirmed [53–55]. Regarding Very poor or Poor trajectories in all domains, patients scored higher on Neuroticism and trait anxiety and lower on Extraversion compared to other trajectories. Different results were found for Conscientiousness and Agreeableness. Surprisingly, except for high education in environment trajectory, no sociodemographic (e.g., female sex) and clinical characteristics were found as risk factors for impaired QOL, which is contrary with earlier research [12, 15, 17, 18].

A major strength and study implication is that it identified patients at risk for impaired QOL. This knowledge will help clinicians to screen patients in an early stage, for example, on the emergency department or department of surgery, by using the Psychosocial Screening Instrument for physical Trauma patients (PSIT) [56]. In addition, the trajectories seemed to be stable during 12 months after trauma. However, RMLCA evaluates characteristics of individuals and not whether a change in development of symptoms is statistically significant. Therefore, interpretation of the course of trajectories can be evaluated using repeated-measures ANOVA or mixed-models ANOVA (in case of > two groups). The fact that most identified trajectories did not involve change over time suggests that QOL at baseline is almost the same 12 months after trauma. Therefore, patients can also be asked about their QOL almost directly after trauma, as this implies QOL 12 months post-trauma. Then, patients can be treated to prevent a psychological disorder. Concerning trajectories of social relationships, patients seemed to rate their social relationships better than the norm scores. A reason could be that trauma patients, who are dependent on others, rate their QOL better when they experienced being supported by their relatives than patients who are not dependent of others and receive less support. Unfortunately, Hawthorn et al. (2006) did not provide norm scores for overall QOL and general health [29]. However, trajectories for overall QOL and general health were indicated based on the labels provided for the other domains. Also, to the best to our knowledge, this was the first study that examined QOL domains after a physical trauma. Because of inconclusive results regarding recovery trajectories, more research is needed that examines QOL domains. Also, pre-injury HRQOL [57] or HS [58, 59] was likely to be a predictor of post-trauma HRQOL and HS. It is still unclear whether pre-injury QOL could be a predictor for post-trauma QOL. In addition, future research could also focus on sociodemographic and clinical characteristics to determine which characteristics mostly influence QOL trajectories and to clarify inconsistent results.

Some limitations must be taken into account. First, as this hospital is a level-1 trauma center, only severely injured patients were included [1]. This may limit the generalizability to other severely injured patients from other level-1 trauma centers or less severely injured patients from level-2 or -3 hospitals. Also, the observed differences in characteristics of responders and non-responders suggest that selection bias might have occurred. Second, the response rate was 27%. Main reason to decline participation was that patients were not interested, because they did not experience any physical or psychological problems after trauma. In contrast, participation could be difficult, because patients could be faced with their problems or (physical) limitations. Furthermore, concerning our dropout rates, it is likely that patients who were fully recovered were probably less interested to complete follow-up measurements compared to patients who still experienced problems with functioning. This could also be the reason for the sparse data in the cross tables comparing the ASD diagnoses between the trajectory classes. Since this sparsity resulted in extremely large odds ratios, we expressed these associations using the phi coefficient. In addition, two kinds of missingness were taken into account. First, missing values on the dependent variables (i.e., WHOQOL-BREF) were handled through full information maximum likelihood estimation using Latent Gold software. This method is appropriate when one or two follow-up measurements are missing from a participant. The second method focussed on single missing item scores of the IES-R and the HADS, which were imputed with individual subscale means when at least half of the subscale items were answered [34, 44, 45]. However, overestimation of item variation and a lower Cronbach's alpha of the scale from that item could occur [60]. Furthermore, the risk factors for QOL were interpreted in terms of correlation and this interpretation did not imply causation [61]. Another limitation is that this study was largely based on self-reported questionnaires. A PTSD diagnosis could not solely rely on self-report questionnaire, as a consultation from a health psychologist or psychiatrist is needed to be diagnosed with PTSD. Therefore, interpretation of such a diagnosis must be done with caution. Finally, no significant changes in trajectories were observed during 12 months post-injury. Since the strength of RMLCA had been to identify how many patterns of responses (i.e., trajectories of QOL) are present in the data and how these patterns are characterized over multiple time points [24]. Therefore, instead of screening patients on risk factors (e.g., ASD, anxiety, depressive symptoms, or personality traits), HCPs could ask them about their needs, perspectives, and satisfaction with QOL almost directly after trauma (at baseline). Future research could focus on the need and the impact of further additional care, from a social worker or registered health psychologist, on patients’ recovery and QOL [62].

## Conclusion

The present study demonstrates that psychological characteristics influence the development of QOL during 12 months after trauma. These findings can enable HCPs to identify patients at risk of impaired QOL. Then, they can offer patient-centered care and, subsequently, patients’ QOL after trauma could be improved.

## Supplementary information

Below is the link to the electronic supplementary material.

Electronic supplementary material 1 (DOCX 29 kb)
