# Supplementary material for: Psychological risk factors that characterize the trajectories of quality of life after a physical trauma: a longitudinal study using latent class analysis
Source: Qual Life Res. 2021 Jan 14;30(5):1317–35. doi: 10.1007/s11136-020-02740-x (PMC8068651; doi:10.1007/s11136-020-02740-x)
Supplement: Supplementary file 1 — Electronic supplementary material 1 (DOCX 29 kb) [file 11136_2020_2740_MOESM1_ESM.docx]

### Supplemental Table 1. Missing sum scores on each quality of life domain for every measurement during 12 months follow-up.

| **Domains** | **Baseline** | **3 mo FU** | **6 mo FU** | **9 mo FU** | **12 mo FU** |
| --- | --- | --- | --- | --- | --- |
| Physical health | 2 (0.7%) | 53 (19.9%) | 61 (22.8%) | 74 (27.7%) | 76 (28.5%) |
| Psychological health | 0 (0%) | 54 (20.2%) | 61 (22.8%) | 74 (27.7%) | 75 (28.1%) |
| Social relationships | 1 (0.4%) | 55 (20.6%) | 62 (23.2%) | 74 (27.7%) | 76 (28.5%) |
| Environment | 1 (0.4%) | 53 (19.9%) | 61 (22.8%) | 74 (27.7%) | 75 (28.1%) |
| Overall QOL and general health | 0 (0%) | 54 (20.2%) | 62 (23.2%) | 74 (27.7%) | 75 (28.1%) |

Number of missing domain scores with percentages are presented. *Abbreviations*: mo: months, FU: follow-up, QOL: quality of life
